# Flow Diversion for Ruptured Tiny Internal Carotid Artery Aneurysm in Patient Allergic to Acetylsalicylic Acid: Case Report and Literature Review

**DOI:** 10.1055/a-2707-0515

**Published:** 2025-10-06

**Authors:** Kalousek Vladimir, Ozretić David, Bilandzic Josko, Rotim Kresimir, Culo Branimir

**Affiliations:** 1 Department of Radiology, University Hospital Center Sestre Milosrdnice, Zagreb, Croatia; 2 Department of Diagnostic and Interventional Neuroradiology, University Hospital Center Zagreb, Zagreb, Croatia; 3 Department of Neurosurgery, University Hospital Center Sestre Milosrdnice, Zagreb, Croatia; 4 Department of Anatomy and Physiology, University of Applied Health Sciences, Zagreb, Croatia; 5 Department of Anatomy and Physiology, J. J. Strossmayer University of Osijek Faculty of Medicine, Osijek, Croatia

**Keywords:** blood-blister aneurysm, flow diverter, antiplatelet monotherapy, subarachnoid hemorrhage

## Abstract

**Introduction:**

Female patient, age 50, allergic to acetylsalicylic acid (ASA) presented to the emergency department of our institution with spontaneous and severe headache.

**Case Report:**

Emergent brain MSCT and CTA scan showed subarachnoid hemorrhage with aneurysm in the C7 segment of left internal carotid artery (ICA). Prasugrel monotherapy was started and she underwent endovascular aneurysm occlusio. Small, atypically shaped aneurysm was found at the origin of anterior choroidal artery (AChA). Flow diverter stent was placed in the left C7 segment. One single coil was deployed in the sac. She was discharged without any neurological sequelae with prasugrel monotherapy. Two years after the procedure, aneurysm was completely occluded with normal flow in left ICA and its branches.

**Discussion:**

Here, we describe case of blood-blister like aneurysm (BBA) at the origin of left AChA. There is still no consesus regarding optimal treatment strategy for BBAs. Our experience shows it is possible to treat BBA with flow diversion even in the acute setting and near origins of ICA branches. Flow diversion needs to be reinforced with aneurysm coiling in the case of ruptured aneurysm. Due to patient's ASA allergy, we opted for prasugrel monotherapy which proved to be both safe and effective antiplatelet therapy after flow diverter placement.

**Conclusion:**

To the best of our knowledge this is first published case in which coiling with flow diversion was used to treat BBA at the branching point of supraclinoid ICA in a patient allergic to ASA.

## Introduction

Female patient, age 50, presented to the emergency department of our institution with sudden, spontaneous, severe pulsating headache. She described headache as worst in her life. Headache was accompanied by nausea and photophobia, but without vomiting and loss of consciousness. Nuchal rigidity was noticed in her neurological exam, without any other neurological signs (HH 1). Past medical history was notable for epilepsy which was controlled by lamotrigine monotherapy. She was allergic to acetylsalicylic acid (ASA).

## Case Report

**Fig. 1:**
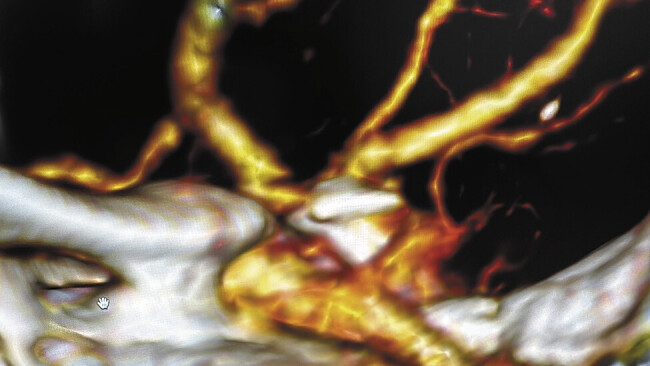
CTA reconstruction showing blood-blister like aneurysm located in the C7 segment of left ICA.

**Fig. 2:**
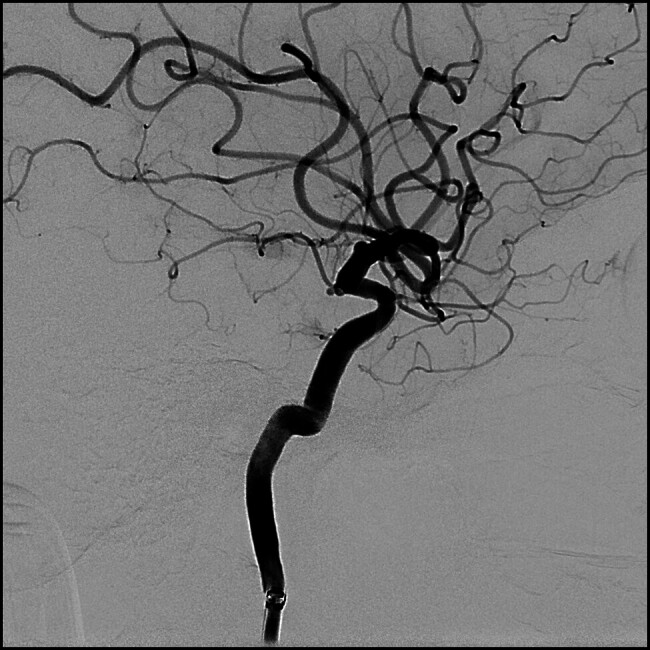
Blood-blister like aneurysm located in the C7 segment of left ICA near AChA origin.

**Fig. 3:**
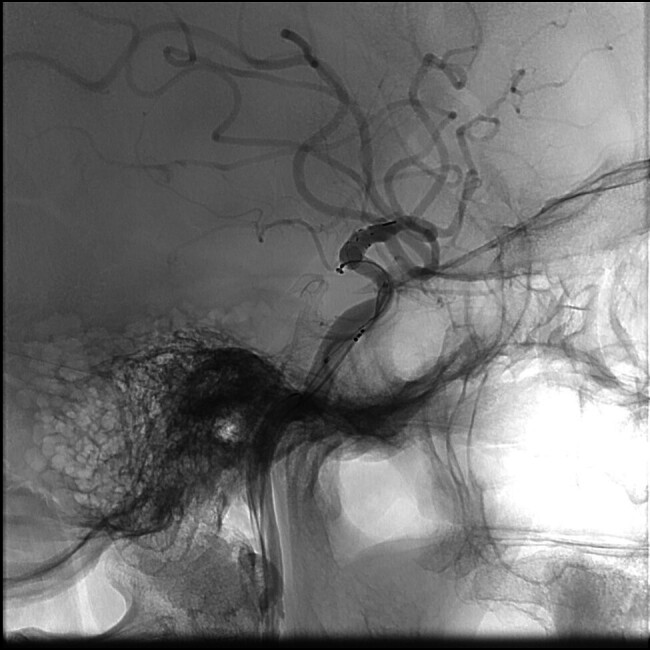
Final microcatheter position achieved with semi-deployment of flow diverter and with single loop of coil inside aneurysmal sac.

**Fig. 4:**
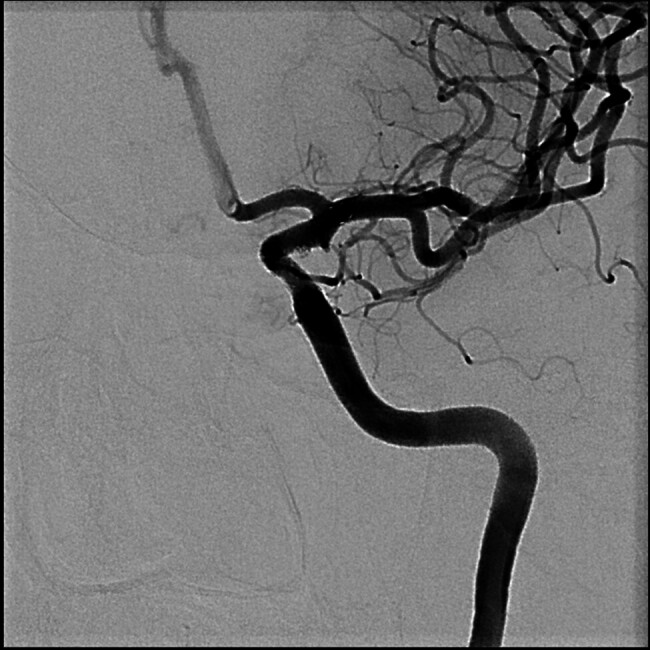
Occluded BBA aneurysm with patent ICA and branching arteries, AP view.

**Fig. 5:**
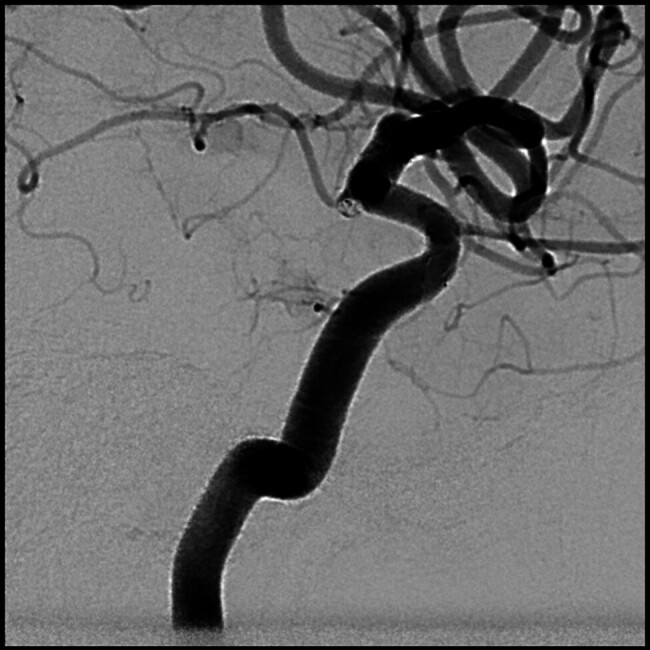
Occluded BBA aneurysm with patent ICA and branching arteries, lateral view.

**Fig. 6:**
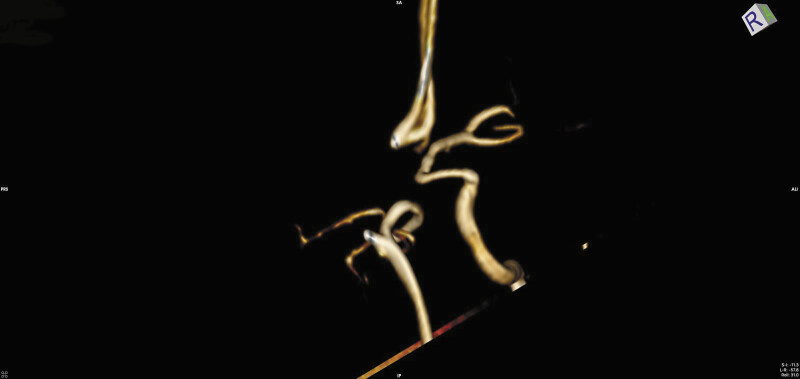
Two year follow-up MRA showing patent flow diverter in the left ICA without aneurysm recurrence, oblique view.

**Fig. 7:**
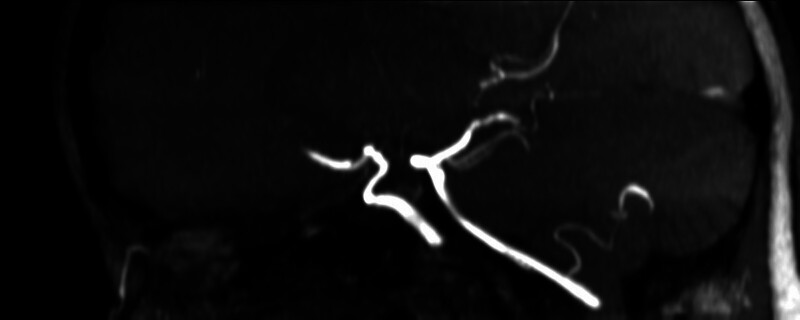
Two-year follow-up MRA showing patent flow diverter in the left ICA without aneurysm recurrence, lateral view.

**Fig. 8:**
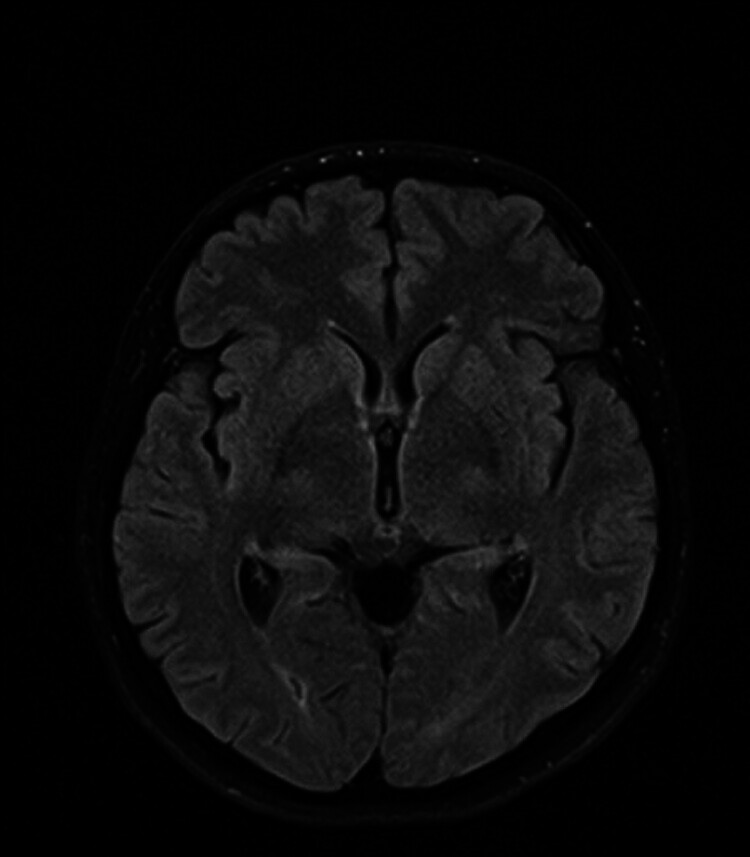
Two-year follow-up brain MR showing no signs of ischemia in the left ICA irrigation zone.

Emergent brain MSCT scan showed subarachnoid hemorrhage (SAH) in basal cisterns, suggestive of aneurysm rupture (Fisher 2). CT angiography confirmed aneurysm in the C7 segment of left internal carotid artery (ICA). Subsequently, she underwent emergent cerebral DSA and endovascular aneurysm occlusion under general anesthesia. Atypically shaped 2 mm aneurysm, highly reminiscent of blood-blister aneurysm (BBA) was found at the origin of anterior choroidal artery (AChA; Figs. 1 and 2 ). Prior to endovascular procedure she was given antiplatelet prasugrel monotherapy due to her ASA allergy. During the endovascular procedure, a flow diverter stent (FRED, MicroVention) was placed in the C7 segment of left ICA, completely covering the aneurysm alongside AChA origin. FRED allowed us to position its single-layer strouts in the M1 segment while double layer portion started below the carotid terminus, covering the aneurysm's orifice. We wanted to cover the aneurysm neck with its double-layer part. At the same time, we wanted to reduce the risk of distal ischemia with a single-layer part going over carotid terminus into M1 segment across A1 origin Variation of coil-patch technique and microcathether jailing were performed. Microcathether Echelon 10 (Medtronic) was steam shaped which allowed us to position it in a way which enabled flow diverter stent to push the microcathter toward aneurysmal sac during opening ( Fig. 3 ). Afterward, one single coil was deployed in the aneurysm sac which was followed by flow diverter full opening ( Figs. 4 and 5 ). Since the aneurysm was protected, we could maintain the prasugrel monotherapy without risking aneurysm rerupture. Postprocedural course was complicated by right femoral artery occlusion at the puncture site, which was treated with embolectomy by vascular surgeon. During the immediate postprocedural period, she was prophylactically treated with nimodipine. Transcranial Doppler data, including Lindegaard ratio, were not suspicious for vasospasm development. She was discharged without any neurological sequelae with prasugrel monotherapy. At her last follow-up exam, 2 years after the procedure, aneurysm was completely occluded (RR 1) with normal flow in left ICA and its branches ( Figs. 6 – 8 ). She remained neurologically intact.

## Discussion

Here, we describe case of blood-blister like aneurysm (BBA) at the origin of left AChA. Blood-blister aneurysms typically arise from nonbranching sites of ICA and account for up to 7% of ICA aneurysms. 1 According to some autopsy studies BBA is presumed to be type of pseudoaneurysm. Currently, it is proposed that a penetrating ulcer in the internal elastic lamina enables blood flow to form a broad based, localized arterial wall defect. 2 Anterolateral side of ICA and especially branching points are a highly unusual position for BBA. However, BBAs have been found at sites other than nonbranching supraclinoid ICA, such as anterior communicating artery described by Morris and Brophy. BBA poses a significant challenge for both diagnostics and treatment. Due to its small size and nonsacular shape this type of aneurysm might be one of possible causes of angiogram negative SAH. 3 Additionally, they are known to grow and change shape in much shorter intervals than sacular aneurysms. 4 There is still no consesus regarding optimal treatment strategy for BBAs. According to one systematic review, surgical morbidity, and mortality are 21 and 17%, respectively. Endovascular treatment appears safer but still has high morbidity and mortality rates of 3.4 and 11.5%, respectively. 5 Various surgical strategies have been reported, such as clipping, aneurysm wrapping, traping with or without bypassing. Surgical procedures are commonly complicated by arterial lacerations, residual ICA stenosis with potential to cause ischemia in the distal ICA teritory. 6 Most commonly employed endovascular treatment strategies include stent-assisted coiling, stent within stent technique, or covered stent placement. 2 Some authors believe that endovascular ICA trapping after successful balloon occlusion test is the only durable option for BBAs, since all other treatment options lead to unacceptably high regrowth and rebleeding rates. 7 Our experience shows it is possible to treat BBA with flow diversion even in the acute setting and near the origins of ICA branches. As we have shown previously, a flow diverter can safely be used in the communicating segment of ICA with low risk of inducing ischemia in ICA branches' territory. 8 However, we believe flow diversion needs to be reinforced with aneurysm coiling in the case of a ruptured aneurysm, as it is imperative to immediately exclude the aneurysm from the circulation to prevent rebleeding. Based on available data antiplatelet therapy seems to be safe in the setting of aneurysmal SAH (aSAH) after stent or flow diverter placement. Due to patient's ASA allergy, we opted for prasugrel monotherapy which proved to be both safe and as effective as dual antiplatelet therapy after flow diverter placement. 9 10 Date given by our colleagues from the cardiology department helped us to reach our decision to use prasugrel monotherapy. According to their literature prasugrel was more potent in preventing stent thrombosis without raising incidence of major bleeding when compared with other antiplatelet medications. 11 According to a recently published meta-analysis, it reduces risk of cerebral vasospasm and delayed cerebral ischemia. 12 However, in some studies patients receiving dual antiplatelet therapy after aSAH had higher rate of external ventricular drain related hemorrhage. 13

## Conclusion

To the best of our knowledge, this is the first published case in which coiling with flow diversion was used to treat BBA at the branching point of the supraclinoid ICA in a patient allergic to ASA. BBAs remain complicated neurosurgical entity due to high risk of rapid growth, rebleeding after rupture, as well as high treatment risk. Still, we believe further clinical research is needed on this topic as we cannot draw definitive conclusions based on our very limited experience.

## References

[1] Kim B M Chung E C Park S I Choi C S Won Y S Treatment of blood blister-like aneurysm of the internal carotid artery with stent-assisted coil embolization followed by stent-within-a-stent technique. Case report J Neurosurg 2007 107 06 1211 1213 18077959 10.3171/JNS-07/12/1211

[2] Lee B H Kim B M Park M S Reconstructive endovascular treatment of ruptured blood blister-like aneurysms of the internal carotid artery J Neurosurg 2009 110 03 431 436 19046039 10.3171/2008.7.JNS08257

[3] Morris T C Brophy B P Blister-like aneurysm of the anterior communicating artery J Clin Neurosci 2009 16 08 1098 1100 19467872 10.1016/j.jocn.2008.10.025

[4] Sim S Y Shin Y S Cho K G Blood blister-like aneurysms at nonbranching sites of the internal carotid artery J Neurosurg 2006 105 03 400 405 16961134 10.3171/jns.2006.105.3.400

[5] Gonzalez A M Narata A P Yilmaz H Blood blister-like aneurysms: single center experience and systematic literature review Eur J Radiol 2014 83 01 197 205 24231267 10.1016/j.ejrad.2013.09.017

[6] Park J Blood blister-like aneurysm with rupture point close to origin of anterior choroidal artery J Korean Neurosurg Soc 2014 56 06 500 503 25628811 10.3340/jkns.2014.56.6.500 PMC4303727

[7] Park J H Park I S Han D H Endovascular treatment of blood blister-like aneurysms of the internal carotid artery J Neurosurg 2007 106 05 812 819 17542524 10.3171/jns.2007.106.5.812

[8] Josko B Kresimir R Ante R Nina R Branimir C Vladimir K Pure arterial malformation of the fetal PCA treated with flow diverter stent-case report and literature review Interv Neuroradiol 2024 15910199241272602 10.1177/15910199241272602 PMC11571153 39113486

[9] Hohenstatt S Saatci I Jesser J Prasugrel single antiplatelet therapy versus aspirin and clopidogrel dual antiplatelet therapy for flow diverter treatment for cerebral aneurysms: a retrospective multicenter study AJNR Am J Neuroradiol 2024 45 05 592 598 38453414 10.3174/ajnr.A8163 PMC11288545

[10] de Castro-Afonso L H Machado J P Nakiri G S Two year follow-up of distal unruptured intracranial aneurysms treated with a surface modified flow diverter under prasugrel monotherapy J Neurointerv Surg 2024 16 11 1163 1166 37524519 10.1136/jnis-2023-020397

[11] DigiMed Bayern Consortium Krüger N Krefting J Kessler T Ticagrelor vs prasugrel for acute coronary syndrome in routine care JAMA Netw Open 2024 7 12 e2448389 39621344 10.1001/jamanetworkopen.2024.48389 PMC11612834

[12] Lee K S Lee C Dhillon P S Antiplatelet therapy in aneurysmal subarachnoid hemorrhage: an updated meta-analysis Neurosurg Rev 2023 46 01 221 37665377 10.1007/s10143-023-02120-2 PMC10477151

[13] Qoorchi Moheb Seraj F Mirbolouk M H Vaezi M Safety of dual antiplatelet therapy in the acute phase of aneurysmal subarachnoid hemorrhage: a propensity score-matched study Neurosurg Focus 2023 55 04 E10 10.3171/2023.7.FOCUS23376 37778032

